# Use and inhalation technique of inhaled medication in patients with asthma and COPD: data from a randomized controlled trial

**DOI:** 10.1186/s12931-018-0936-3

**Published:** 2018-12-03

**Authors:** Claudia Gregoriano, Thomas Dieterle, Anna-Lisa Breitenstein, Selina Dürr, Amanda Baum, Sabrina Maier, Isabelle Arnet, Kurt E. Hersberger, Jörg D. Leuppi

**Affiliations:** 1 University Clinic of Medicine, Cantonal Hospital Baselland, Rheinstrasse 26, CH - 4410 Liestal, Switzerland; 2 Department of Pharmaceutical Sciences, University of Basel, Basel, Switzerland; 3 Faculty of Medicine, University of Basel, Basel, Switzerland

**Keywords:** Asthma, Pulmonary disease, chronic obstructive, Inhalation technique, Dry powder inhalers, Metered dose inhalers, Quality of life, lung function

## Abstract

**Background:**

The burden of asthma and COPD among patients is high and people affected are frequently hospitalized due to exacerbations. There are numerous reasons for the lack of disease control in asthma and COPD patients. It is associated with non-adherence to guidelines on the part of the health care provider and with poor inhalation technique and/or non-adherence to the prescribed treatment plan by the patient. This study aims to present data on inhaler technique and its impact on quality of life (QoL) and symptom control in a typical population of patients with chronic lung disease from a randomized controlled trial on medication adherence.

**Methods:**

For this cross-sectional analysis, 165 asthma and COPD patients were analyzed. Correct application of inhaler devices was tested using pre-defined checklists for each inhaler type. QoL and symptom control were investigated using COPD Assessment Test (CAT) and Asthma Control Test (ACT). Spirometry was used to measure forced vital capacity (FVC) and forced expiratory volume in one second (FEV_1_).

**Results:**

Overall, incorrect inhalation technique ranged from 0 to 53% depending on the type of inhaler. COPD patients with incorrect device application had a higher CAT sum score compared to those with a correct device application (*P* = .02). Moreover, COPD patients with incorrect device application were more likely to suffer from cough (*P* = .03) and were more breathless while walking uphill or a flight of stairs (*P* = .02). While there was no significance found in asthma patients, COPD patients who used their devices correctly had a significantly better mean FEV_1_% predicted at baseline compared to those who applied their devices incorrectly (*P* = .04).

**Conclusions:**

Correct inhalation of prescribed medication is associated with improved health status and lung function. These findings should encourage health professionals to provide instructions on correct inhalation technique and to regularly re-evaluate the patients’ inhalation technique.

**Trial registration:**

ClinicalTrials.gov: NCT0238672, Registered 14 February 2014.

## Background

Asthma and Chronic Obstructive Pulmonary Disease (COPD) are chronic respiratory diseases that are highly prevalent in the overall population [1].

To date, asthma and COPD are not curable but treatable diseases of the respiratory system. Nevertheless, the burden of each disease among patients is high and patients may be frequently hospitalized due to exacerbation. Despite effective therapy options and evidence-based guidelines developed in recent years, disease control continues to be suboptimal in patients with these two chronic obstructive lung diseases.

There are numerous reasons for the lack of disease control in asthma and COPD patients. One important reason is the incorrect application of inhaler devices, which is associated with worsened health outcomes, such as increased risk of hospitalization and an insufficient diseases control [2–4].

Inhaled medication play a key role in the treatment of asthma and COPD patients. This application way has the advantage to deliver the drug directly into the airways. Therefore, high local concentrations can be achieved with a reduced risk of systemic side effects [5]. However, a variety of different sequential steps are necessary to achieve a correct application of these devices. Incorrect performance of one or more steps can substantially reduce the delivery of the administrated substance and consequently the effectiveness and safety of the medication [6]. Numerous studies have shown that 50–80% of the investigated patients do not use their inhaler devices correctly [7–10]. They often overestimate their inhalation technique or they are not even aware that they are using their inhaled medication incorrectly [11].

Therefore, as also recommended in national and international guidelines, inhalation technique should be assessed on a regular basis, in order to be able to correct the application if necessary.

This cross-sectional analysis aims to present data on inhaler technique and its impact on quality of life and symptom control in a typical population of patients with chronic lung disease from the Adherence-Trial [12]. The longitudinal Adherence-Trial was designed to investigate the effect of a patient-tailored intervention with daily alarm clock and reminder in form of phone calls on adherence to inhaled therapy in asthma and COPD patients and to determine the resulting effect on exacerbations and quality of life [12].

## Methods

### Study design

**Fig. 1:**
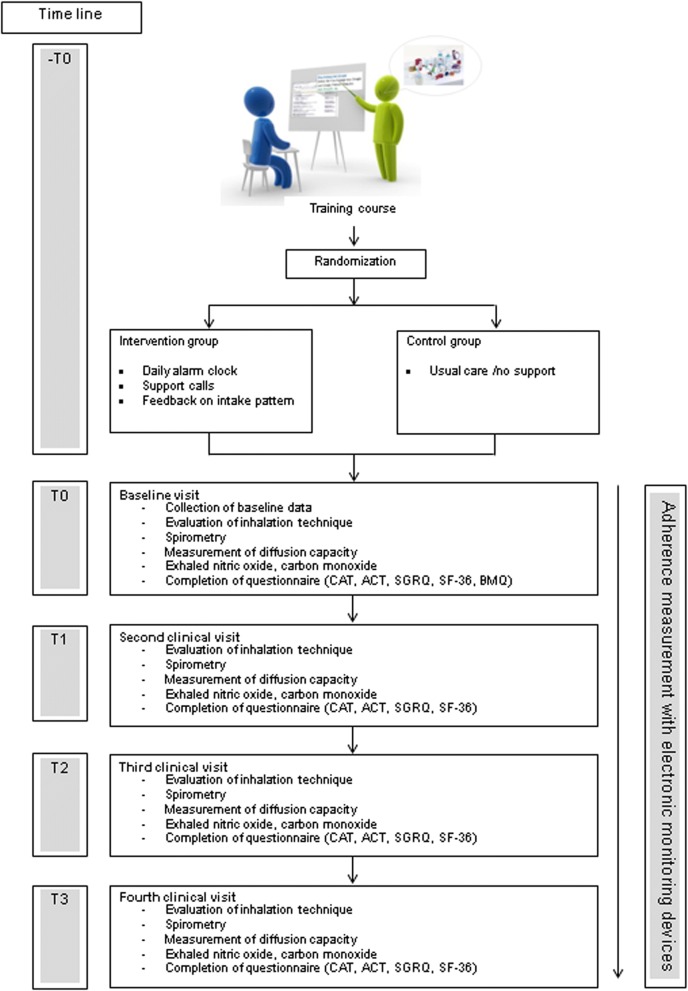
Diagram for detailed study procedure

The Adherence-Trial was a single-blind randomized controlled trial. The study details have been published previously [12]. In brief, adherence to inhaled medication was analyzed over a six-month period in patients with asthma and/or COPD who experienced at least one exacerbation within the previous year. The study was conducted in an ambulatory setting between January 2014 and March 2017. The investigator obtained written consent from patients confirming their willingness to participate in the study. Adherence was measured using electronic data capture devices, which save date and time of each inhalation device actuation and transfer these data daily via wireless-connection to a web-based database. All participants took part in a training course before the baseline visit. The goal of the training course was to provide refresher training on inhalation techniques, to ensure that all participants were at the same level of knowledge about their disease and used their medication correctly. The training began with a brief introduction about asthma and COPD. Afterwards, the most frequently used devices were presented and briefly demonstrated. Common mistakes and problems associated with the use of the devices were explained. The correct use of the individual devices was demonstrated in a short film (produced by the “Deutsche Atemwegsliga” Bad Lippspringe, Germany) [13].  At the end of the training, participants were given the opportunity to ask questions concerning the devices. After the training course, patients were randomly assigned to either the intervention or the control group. The intervention group received a daily alarm clock and reminder in form of support calls in case medication was not taken as prescribed or the use of rescue medication doubled. During the study, participants were assessed every two months in the form of clinical visits. The detailed study procedure is illustrated in Fig. 1. Prior to initiation the trial was approved by the local ethics committee (registry number: EK-269/13) and registered (ClinicalTrials.gov: NCT02386722).

### Measurements

#### Sociodemographic factors

Sociodemographic variables such as age, gender, marital status and education level were obtained by a generic questionnaire at the baseline visit. Furthermore, smoking status, as well as pack years (py) and body mass index (BMI) were recorded during this visit. In addition, disease-related questions like allergies, number of exacerbations during the previous 12 months including treatments with antibiotics, treatment with systemic corticosteroids, emergency department attendance and hospitalizations were asked. If patients were not able to provide information about the events, the treating physician was contacted. Moreover, the current inhaled medication was recorded. The respective diagnosed lung diseases (asthma, COPD or ACO) were taken from previous medical reports and were not redefined or diagnosed again for this study.

#### Lung function

Spirometry was used to measure forced vital capacity (FVC) and forced expiratory volume in one second (FEV_1_). To obtain information about pulmonary gas transfer, diffusing capacity of the lung for carbon monoxide (DLCO) was measured. All tests were performed according to the guidelines of the European Respiratory Society [14].  The device EasyOne Pro (ndd Medizintechnik AG, Zürich, Switzerland) was used.

#### Evaluation of the device application

To detect false device application, each patient was asked to demonstrate the inhalation technique with all prescribed devices to the investigator by using a placebo device. As prescribed in the study protocol, correct use was assessed by using pre-defined checklists for each inhaler type based on user guidelines and instruction package inserts from the manufacturers. Inhaler technique was accepted as correct, when every step controlled in the checklist was performed accordingly. The technique was defined as wrong if one or more steps were done incorrectly. For each incorrect step, participants received a score of “0” whereas each correct application was valued as “1”. A critical error was defined as incorrect completion of a step that significantly reduces or totally inhibits drug delivery and thus impacts the effectiveness of the delivered drug [15]. Evaluation of the device application was assessed by a trained pharmacist. To standardize the observation of errors as much as possible, the same person (pharmacist) always evaluated the device application. Further, as part of the training, the pharmacist regularly watched inhaler videos [13] for the relevant devices types, in order to gain proficiency in the evaluation. The evaluation was performed by a single observer (intra-observer variability).

#### Asthma control test (ACT)

The ACT questionnaire was applied in order to assess asthma control. The validation of the questionnaire showed that scores calculated from the ACT are reliable and valid, and that the test has the ability to screen for patients with poorly controlled asthma. It represents a clinically validated measure of asthma control that is simple to administer to assess the level of asthma control with or without the use of lung function tests [16]. The ACT score ranges from 5 to 25. Values ranging between 5 and 15 indicate “very poor controlled asthma”, those from 16 to 19 “not well controlled asthma” and values varying from 20 to 25 signify “well controlled asthma” [16].

#### Impact of COPD symptoms

The health status of COPD patients was measured using the CAT (COPD Assessment Test). The CAT is a validated, disease-specific eight-item questionnaire on a sematic six-point differential scale. It is developed to measure the impact of the lung disease on patients’ health status. Scores from 0 to 10, 11–20, 21–30 and 31–40 represent a “low”, “medium”, “high” and “very high” impact of the disease on a person’s health status [17].

#### Assessment of health-related quality of life

Health-related quality of life was assessed using the St. George Respiratory Questionnaire (SGRQ), a standardized questionnaire developed for measuring impaired health and perceived well-being (“quality of life”) in patients with asthma and COPD. Scores range from 0 to 100, with the highest scores indicating the poorest level of respiratory health as well as maximal disability [18, 19].

### Sample size calculation

Power calculation is based on “time to next exacerbation”, which was defined as primary endpoint of the overall Adherence-Trial. We expected an assumed endpoint reduction of 60% (12/30), with 12% (8/70) of patients experiencing an exacerbation in the intervention group. Assuming a sample size of 70 participants for each study group, there is a power of 80% to detect a hazard ratio (HR) of 0.36 based on a 1-tailed test with a 5%-significance level, since only a decrease of the exacerbation-risk is of interest and expected. For each study group 7 additional participants were added to account for dropouts. Therefore, a minimum of 154 participants were included in this study.

### Statistical analysis

Data were analyzed using the SPSS software package (version 23, IBM, Germany). Statistical significance was set at the 5% level. Data are presented as mean ± standard deviation (SD) or number and percentage (%). To check the data for normal distribution, the Shapiro-Wilk test was used. For two unrelated parametric conditions, the independent t-test was applied, while for two unrelated non-parametric conditions, the Mann-Whitney test was calculated. In order to investigate the relationship between categorical variables, the Pearson’s chi-square test was applied. Poisson regression was used to quantify exacerbation count with respect to the participants’ device handling before study start.

## Results

**Fig. 2:**
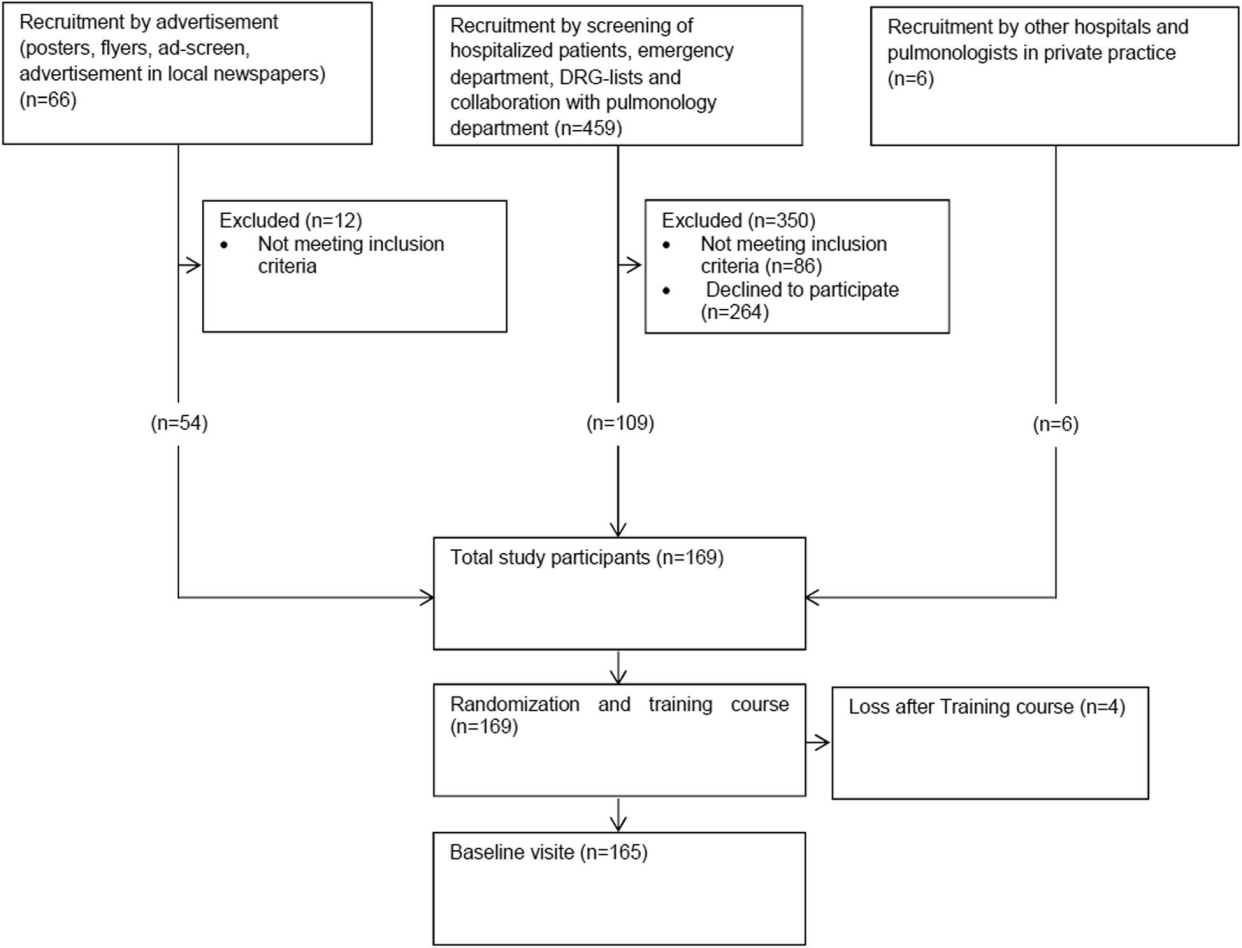
Flow chart of study

A total of 169 asthma and COPD patients were recruited to participate in the study. Four patients withdrew after the training course. Therefore, a total of 165 patients (84 interventions, 81 controls) were assessed and analyzed at baseline (Fig. 2).

### Patients’ characteristics

Patients’ characteristics are presented in Table 1. Approximately 40% of the asthma patients had not well- or poorly controlled asthma at baseline, while less than 30% of the COPD patients showed a high- and very high impact of the disease on their health status.

**Table 1:** Characteristics of the 165 study participants at baseline

Variable	Number(%) or Mean ± SD			
	All (*n* = 165)	Asthma (*n* = 50)	COPD (*n* = 89)	ACO (*n* = 26)
Age	66.8 ± 11.5	61.1 ± 15.1	69.8 ± 8.5	67.2 ± 8.0
Male	106 (64.2)	23 (46)	63 (70.8)	20 (76.9)
Marital status				
Unmarried	19 (11.5)	7 (14)	11 (12.4)	1 (3.8)
Married	104 (63.0)	33 (66)	51 (57.3)	20 (76.9)
Divorced/widowed	42 (25.5)	10 (20)	27 (30.3)	5 (19.2)
Highest level of education at school				
Primary school	27 (16.4)	6 (12)	18 (20.2)	3 (11.5)
Apprenticeship	97 (58.8)	25 (50)	55 (61.8)	17 (65.4)
Higher professional education	22 (13.3)	7 (14)	11 (12.4)	4 (15.4)
University–entrance Diploma/Commercial college	3 (1.8)	3 (6)	0 (0)	0 (0)
University /College of higher education	16 (9.7)	9 (18)	5 (5.6)	2 (7.7)
Smoking status				
Current smokers	32 (19.4)	5 (10)	24 (27)	3 (11.5)
Non-smokers	37 (22.4)	28 (56)	5 (5.6)	4 (15.4)
Ex-smokers	96 (58.2)	17 (34)	60 (67.4)	19 (73.1)
Pack-years	35.0 ± 34.3	8.0 ± 14.3	58.5 ± 33.0	32.3 ± 35.5
Body mass index [kg/m^2^]	27.2 ± 5.1	26.9 ± 3.9	26.9 ± 5.6	28.5 ± 5.3
GOLD stage (*n* = 115)				
1 (FEV_1_ > 80% predicted)	8 (6.9)		6 (6.7)	2 (7.7)
2 (FEV_1_ 50–80% predicted)	47 (40.9)		33 (37.1)	14 (53.8)
3 (FEV_1_ 30–50% predicted)	46 (40.0)		36 (40.4)	10 (38.5)
4 (FEV_1_ < 30% predicted)	14 (12.2)		14 (15.7)	0 (0)
Lung function parameters				
FEV_1_% predicted	59.9 ± 24.5	80.0 ± 19.9^a^	48.2 ± 20.0	61.9 ± 21.1^b^
FVC % predicted	90.2 ± 19.0	98.7 ± 17.7^a^	85.0 ± 18.4	92.3 ± 17.6^b^
FEV_1_/FVC ratio	51.9 ± 16.7	66.3 ± 12.3^a^	43.7 ± 13.9	52.9 ± 14.3^b^
DLCO % predicted	72.2 ± 21.8	88.9 ± 15.7	61.1 ± 19.2	78.0 ± 17.5
Asthma Control Test (*n* = 76)				
Sum score	19.5 ± 4.5	20.7 ± 3.8		17.15 ± 5.0
Poorly controlled	15 (19.7)	5 (10)		10 (38.5)
Not well-controlled	14 (18.4)	8 (16)		6 (23.1)
Well controlled	47 (61.9)	37 (74)		10 (38.5)
COPD Assessment Test (*n* = 115)				
Sum score	16.1 ± 7		16 ± 7	16.6 ± 7.2
Low impact	25 (21.8)		19 (21.3)	6 (23.1)
Medium impact	58 (50.4)		46 (51.7)	12 (46.2)
High impact	29 (25.2)		22 (24.7)	7 (26.9)
Very high impact	3 (2.6)		2 (2.2)	1 (3.8)
SGRQ (*n* = 165)				
Symptoms score	47.0 ± 24.1	35.9 ± 19.1	50.6 ± 23.6	55.9 ± 27.9
Activity score	48.6 ± 22.3	34.5 ± 21.0	54.9 ± 20.2	54.4 ± 20.0
Impact score	25.6 ± 18.1	19.1 ± 15.4	27.6 ± 17.7	31.1 ± 21.0
Total Score	36.1 ± 18.1	26.6 ± 15.4	39.6 ± 17.2	42.3 ± 19.6
Known allergies	68 (41.2)	33 (66)	17 (19.1)	18 (69.2)
Number of exacerbations in the past 12 months				
1	92 (55.8)	29 (58)	50 (56.2)	13 (50)
2	28 (17)	11 (22)	15 (16.9)	2 (7.7)
3	21 (12.7)	5 (10)	10 (11.2)	6 (23.1)
> 3	24 (14.5)	5 (10)	14 (15.7)	5 (19.2)
Number of antibiotic treatments in the past 12 months				
Never	13 (7.9)	8 (16)	4 (4.5)	1 (3.8)
Once	91 (55.2)	29 (58)	50 (56.2)	12 (46.2)
2–3 times	43 (26.1)	12 (24)	23 (25.8)	8 (30.8)
More than 3 times	18 (10.9)	1 (2)	12 (13.5)	5 (19.2)
Number of systemic corticosteroid treatments in the past 12 months				
Never	77 (46.7)	23 (46)	44 (49.4)	10 (38.5)
Once	44 (26.7)	12 (24)	26 (29.2)	6 (23.1)
2–3 times	19 (11.5)	7 (14)	7 (7.9)	5 (19.2)
More than 3 times	25 (15.2)	8 (16)	12 (13.5)	5 (19.2)
Number of emergency department attendance				
Never	103 (62.4)	40 (80)	48 (53.9)	15 (57.7)
Once	44 (26.7)	8 (16)	28 (31.5)	8 (30.8)
2–3 times	16 (9.7)	2 (4)	11 (12.4)	3 (11.5)
More than 3 times	2 (1.2)	0 (0)	2 (2.2)	0 (0)
Number of exacerbations with hospitalization in the past 12 months				
Never	97 (58.8)	39 (78)	43 (48.3)	15 (57.7)
Once	51 (30.9)	7 (14)	34 (38.2)	10 (38.5)
2–3 times	14 (8.5)	4 (8)	9 (10.1)	1 (3.8)
More than 3 times	3 (1.8)	0 (0)	3 (3.4)	0 (0)

^a^*n* = 49, ^b^*n* = 25, *ACO* Asthma-COPD-overlap, *DLCO* Diffusing capacity of the lung for carbon monoxid, *FEV*_*1*_ Forced expiratory volume in one second, *FVC* Forced vital capacity, *SGRQ* St. George Respiratory Questionnaire

A summary of the prescribed medication at baseline is illustrated in Table 2. The most frequently prescribed inhaled medications were combinations of LABA and ICS (35.5%), followed by LABA (23.0%) and SABA (20.9%). The majority of the participating patients had dual therapy with a combination of two inhaled medications (44.8%) or monotherapy with only one inhaled medication (29.7%).

**Table 2:** Characteristics of the prescribed medication of the 165 study participants at baseline

Variable	Number (%)			
	All (*n* = 165)	Asthma (*n* = 50)	COPD (*n* = 89)	ACO (*n* = 26)
Medication (*n* = 326)				
LABA/ LAMA combinations	21 (6.4)	1 (0.3)	16 (4.9)	4 (1.2)
LABA/ICS combinations	116 (35.5)	44 (13.5)	50 (15.3)	22 (6.7)
LAMA	75 (23)	5 (1.5)	60 (18.4)	10 (3.1)
LABA	23 (7)	3 (0.9)^a^	15 (4.6)	5 (1.5)
ICS	17 (5.2)	8 (2.5)	4 (1.2)	5 (1.5)
SAMA	2 (0.6)	1 (0.3)	1 (0.3)	0 (0)
SABA	68 (20.9)	28 (8.6)	31 (9.5)	9 (2.8)
SABA/SAMA combinations	4 (1.2)	0 (0)	1 (0.3)	3 (0.9)
Number of inhaled medication at baseline				
1	50 (30.3)	17 (34)	27 (30.3)	6 (23.1)
2	71 (43)	26 (52)	37 (41.6)	8 (30.8)
3	42 (25.5)	7 (14)	23 (25.8)	12 (46.2)
4	2 (1.2)	0 (0)	2 (2.2)	0 (0)

*ACO* Asthma-COPD-overlap, *LABA* Long acting beta_2_-agonist, *LAMA* Long acting muscarinic antagonist, *ICS* Inhaled corticosteroid, *SAMA* Short acting muscarinic antagonist; SABA, Short acting beta_2_-agonist

^a^The 3 asthma patients with a LABA-medication also had an ICS medication as part of their treatment plan (in separate devices and not as a combined preparation). Thus, monotherapy with LABA in asthmatic patients did not occur due to the fact that it can increase the exacerbation risk by masking the asthma symptoms (FDA Drug Safety Communication [38])

### Device application considering the different inhaler types

Table 3 shows the quality of device application subdivided into the different inhaler types used by the study participants. Other devices (e.g. Genuair etc.) not specified in Table 3 were not used by the participants and thus not examined. Overall, incorrect inhalation technique ranged from 0 to 53% depending on the type of inhaler. The highest rate of incorrect device application was identified among patients using metered dose inhalers. Followed by those who applied Turbohaler as well as powder inhalation capsules such as Handihaler and Breezhaler. Patients who showed a higher number of correct device applications either used the Discus or the newest powder device Ellipta®. Table 3 shows the number of errors for each investigated device. When following the pre-defined checklists, which varied according to each inhaler type, there were some steps associated with a considerable number of repeated errors. For the metered dose inhaler with or without spacer, the steps “shake the inhaler before actuation” was performed incorrectly in 37% (*n* = 23), while the step “coordination of actuation and inhalation” which regards only the metered dose inhaler without spacer represented a source of error in 20% (*n* = 17) of patients. Also the “deep and slow” inspiration that is needed for a complete and correct inhalation of the aerosol delivered by the metered dose inhaler was one of the most common points that caused problems (17%, *n* = 14). In case of the Turbohaler application, 30% (*n* = 17) of the patients did not ensure that the device was held upright while charging in order to achieve correct dose loading. Furthermore, 9% (*n* = 6) of the patients who applied the Handihaler did not “breathe out completely before the inhalation” and did not “hold breath for at least 5 seconds after inhalation” (9%, *n* = 6). Moreover, 13% (*n* = 5) of the participants using Breezhaler devices showed “multiple squeezing of the pushbutton to pierce the capsule”. It has to be noted that multiple piercing can cause the capsule to break into particles which requires full replacement of the capsule.

**Table 3:** Number of errors after the training course for each device

Variable	Number (%)						
	MDI	MDI-spacer	Discus	Turbo-haler	Handi- haler	Breez- haler	Ellipta®
	(*n* = 60)	(*n* = 24)	(*n* = 31)	(*n* = 57)	(*n* = 66)	(*n* = 40)	(*n* = 22)
At least one error	32 (53)	6 (25)	4 (13)	22 (39)	14 (21)	10 (25)	0 (0)
Handling error							
Not removing/opening the cap^a^	0 (0)^a^	0 (0)^a^	N.A	0 (0)^a^	N.A	-N.A	N.A
Not shaking the device before actuation	20 (24)^a^	3 (13)^a^	N.A	N.A	N.A	N.A	N.A
No upright posture before inhalation	1 (1)	0 (0)	1 (3)	0 (0)	1 (2)	1 (3)	0 (0)
Poor coordination of actuation and inhalation: triggering before or at end of inspiration^a^	17 (20)^a^	N.A	N.A	N.A	N.A	N.A	N.A
Failure to pierce the capsules	N.A	N.A	N.A	N.A	2 (3)^a^	5 (13)^a^	N.A
Incorrect rotation	N.A	N.A	N.A	2 (3.51)^a^	N.A	N.A	N.A
Incorrect inhaler position^a^	0 (0)	0 (0)	0 (0)	17(29.82)^a^	N.A	N.A	N.A
Failure to load	N.A	N.A	1 (3)^a^	N.A	0 (0)^a^	2 (5)^a^	N.A
Failure to open the device	N.A	N.A	0 (0)^a^	N.A	0 (0)^a^	0 (0)^a^	0 (0)^a^
Mouthpiece not enclose tightly with the lips^a^	6 (7)^a^	0 (0)^a^	0 (0)^a^	1 (1.75)^a^	2 (3)^a^	0 (0)^a^	0 (0)^a^
Inhalation error							
Not holding breath for about 5 s after inhalation^a^	6 (7)	1 (4)	1 (3)	2 (3.51)	6 (9)	1 (3)	0 (0)
No complete expiration before inhalation^a^	5 (6)	1 (4)	2 (7)	4 (7.02)	6 (9)	2 (5)	0 (0)
Expiration into the device^a^	N.A	N.A	0 (0)^a^	0 (0)^a^	0 (0)^a^	0 (0)^a^	0 (0)^a^
No deep and slow inspiration	14 (17)	2 (8)	N.A	N.A	N.A	N.A	N.A
No forceful and deep inspiration^a^	N.A	N.A	1 (3)	3 (5.26)	1 (2)	2 (5)	0 (0)
No vibration of the capsule audible, during inhalation	N.A	N.A	N.A	N.A	1 (2)^a^	1 (3)^a^	N.A
No breathing out with pursed up lip technique after inhalation	0 (0)	0 (0)	1 (3)	0 (0)	1 (2)	0 (0)	0 (0)

^a^Critical errors for this device [15].  MDI, metered dose inhaler; N.A., not applicable for the corresponding device

*T*able 4 shows how often inhaler usage of the specified devices was explained by a health care provider at the time it was first prescribed. The education was carried out most frequently by a pulmonologist (45%, *n* = 108) followed by a general practitioner in 36% (*n* = 87) of the cases. Nurses (8%, *n* = 18), pharmacists (4%, *n* = 10) and physiotherapists (3%, *n* = 7) seem not to play an important role in the education of device usage. Six patients indicated to have received education from a general practitioner as well as from a pulmonologist (3%) and further three patients received instruction from a general practitioner and a physiotherapist (1%).

**Table 4:** Inhaler-usage-education before study start

		Previous inhaler usage education	
Device	n	Education	No education
		Number (%)	
Metered dose inhaler	84	65 (77)	19 (23)
With Spacer	24	19 (79)	5 (21)
Without Spacer	60	46 (77)	14 (23)
Discus	31	29 (94)	2 (6)
Turbohaler	57	47 (82)	10 (18)
Handihaler	66	54 (82)	12 (18)
Breezhaler	40	29 (73)	11 (27)
Ellipta®	22	15 (68)	7 (32)

### Relationship between exacerbation history and device application

Table 5 illustrates the exacerbation history of the 12 months before inclusion in relation to the device handling of the participants. The poisson regression indicated that patients with incorrect inhaler handling, had 1.30 (95% CI,1.04 to 1.63) or 30% times more exacerbations compared to participants with correct inhaler handling.

**Table 5:** Exacerbation history in relation to the device handling of the participants

Exacerbation history	Count	1	2	3	4	5	6
Correct inhaler handling (*n* = 104)	Frequency (%)	66 (63)	18 (17)	8 (8)	8 (8)	4 (4)	0 (0)
Incorrect Inhaler handling (*n* = 61)	Frequency (%)	26 (43)	10 (16)	13 (21)	10 (16)	1 (2)	1 (2)

### Comparison of ACT and CAT between patients with correct and incorrect device application at baseline

ACT and CAT sum scores between asthma, respectively COPD patients with correct and incorrect device application are shown in Table 6. Regarding asthma control, no difference was observed between asthma patients with correct and incorrect device application (*P* = .99). In contrast, COPD patients with incorrect device application had a higher CAT sum score compared to those with a correct device application (*P* = .02).

**Table 6:** Comparison of ACT and CAT between patients with correct and incorrect device application at baseline

	Mean (95% CI)		*P* value
Device application	Correct	Incorrect	
ACT total sum score	19.40 (18.04–20.75)	19.61 (17.93–21.28)	.99
CAT total sum score	14.97 (13.48–16.47)	18.07 (15.70–20.44)	.02
ACT- questions (referring to the last 4 weeks):			
Question 1: Prevention to go work/school	3.90 (3.57–4.22)	4.00 (3.58–4.42)	.71
Question 2: Shortness of breath	3.63 (3.23–4.02)	3.54 (3.02–4.05)	.71
Question 3: Asthma symptoms at night	3.75 (3.33–4.17)	4.04 (3.55–4.52)	.47
Question 4: Use of reliever medication	4.13 (3.75–4.50)	4.11 (3.67–4.55)	.65
Question 5: Self-assessment of asthma control	4.00 (3.73–4.27)	3.93 (3.63–4.23)	.51
CAT- questions:			
Question 1: Frequency of cough	2.22 (1.91–2.53)	2.79 (2.35–3.23)	.03
Question 2: Amount of phlegm	2.10 (1.80–2.39)	2.60 (2.11–3.10)	.08
Question 3: Tightness in the chest	1.72 (1.44–2.00)	2.09 (1.61–2.58)	.22
Question 4: Breathlessness while walking up a hill or one flight of stairs	3.18 (2.84–3.52)	3.77 (3.38–4.16)	.02
Question 5: Limitation doing activities at home	1.81 (1.45–2.16)	2.19 (1.70–2.68)	.21
Question 6: Confidence to leave home because of lung conditions	0.56 (0.33–0.78)	0.67 (0.33–1.02)	.66
Question 7: Sleep quality	1.18 (0.91–1.45)	1.60 (1.10–2.11)	.34
Question 8: Amount of energy	2.21 (1.89–2.53)	2.35 (1.91–2.79)	.53

A subgroup analysis of the single ACT and CAT questions between patients with correct and incorrect device application is also illustrated in Table 6. There was no significant difference for all ACT-questions. However, for the CAT questions there was a significant difference in the question referring to the frequency of cough (*P* = .03) and the question assessing the condition of being “breathless while walking up a hill or flight of stairs” (*P* = .02).

### Comparison of lung function parameters between patients with correct and incorrect device application at baseline

**Fig. 3:**
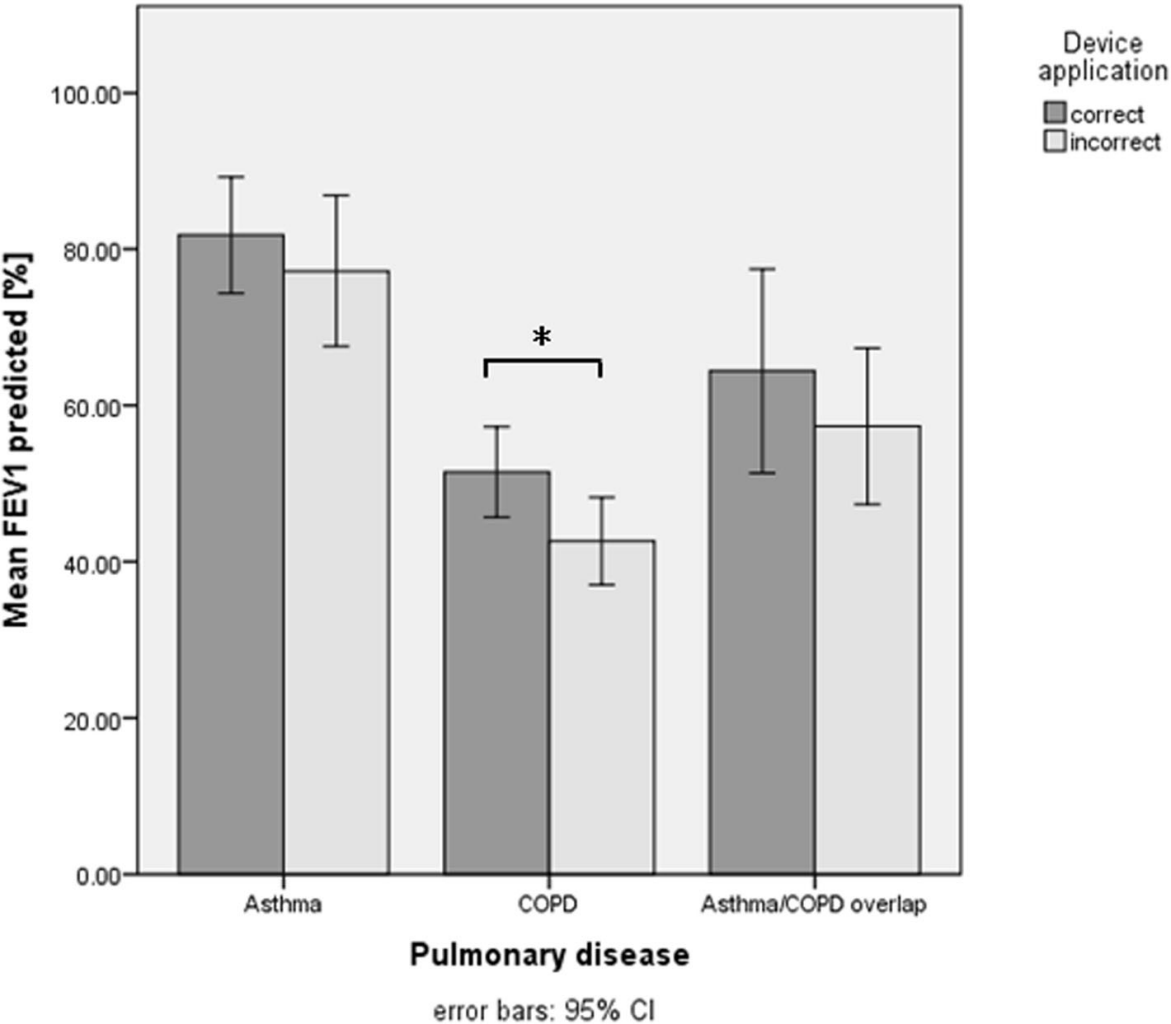
Comparison of the mean FEV_1_% predicted in patients applying their devices corrects an incorrect. FEV_1_, forced expiratory volume in one second

**Fig. 4:**
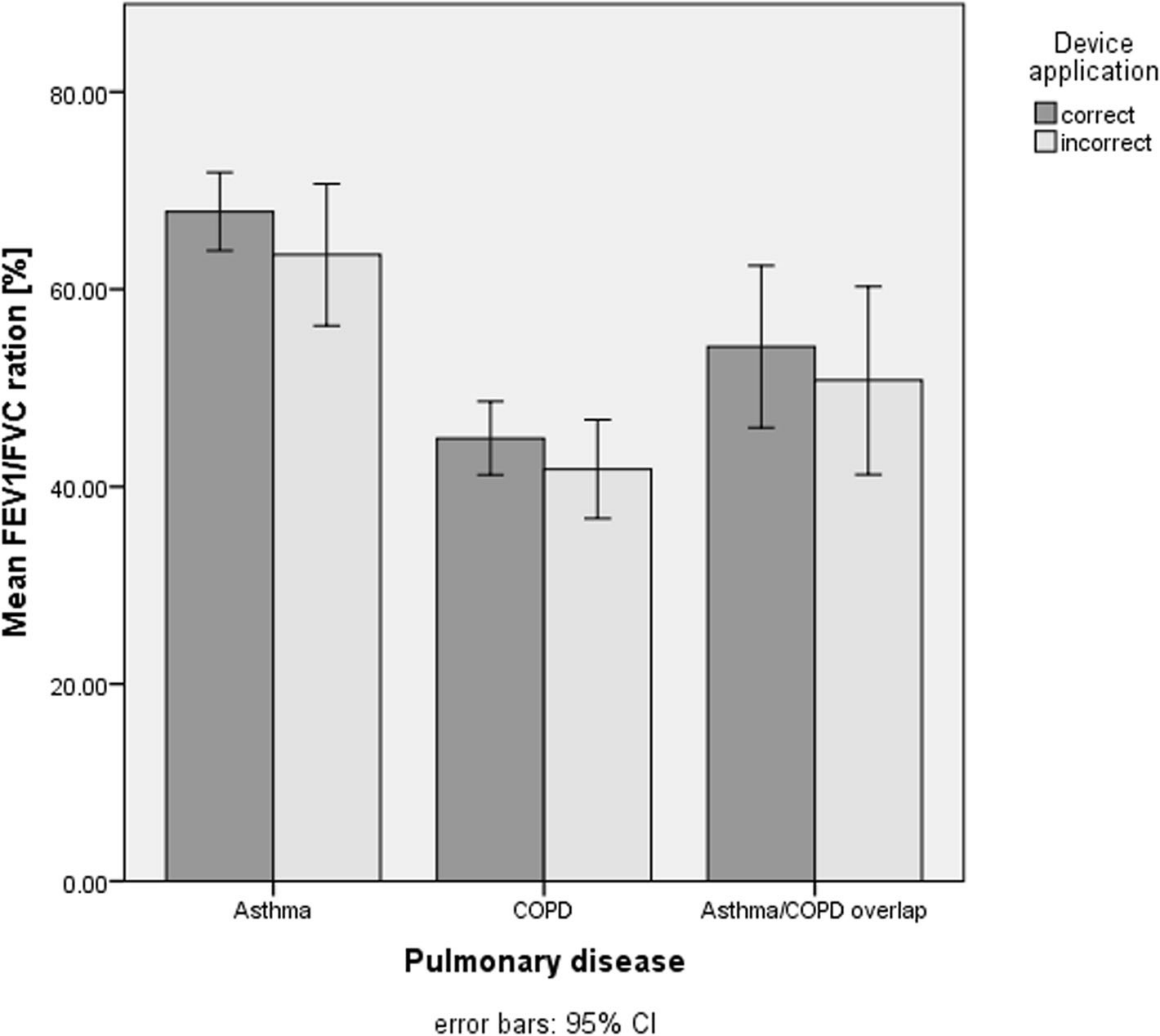
Comparison of the mean FEV_1_/FVC % predicted in patients applying their devices correct and incorrect. FEV_1_, forced expiratory volume in one second; FVC, forced vital capacity

The comparison of the forced expiratory volume in one second (FEV_1_) (Fig. 3) and the Tiffeneau (FEV_1_/FVC) (Fig. 4) between asthma patients with correct and incorrect device application showed no difference. However, COPD patients who applied their devices correctly had a significantly better mean FEV_1_ at baseline compared to those who applied their devices incorrectly (*P* = .04).

**Fig. 5:**
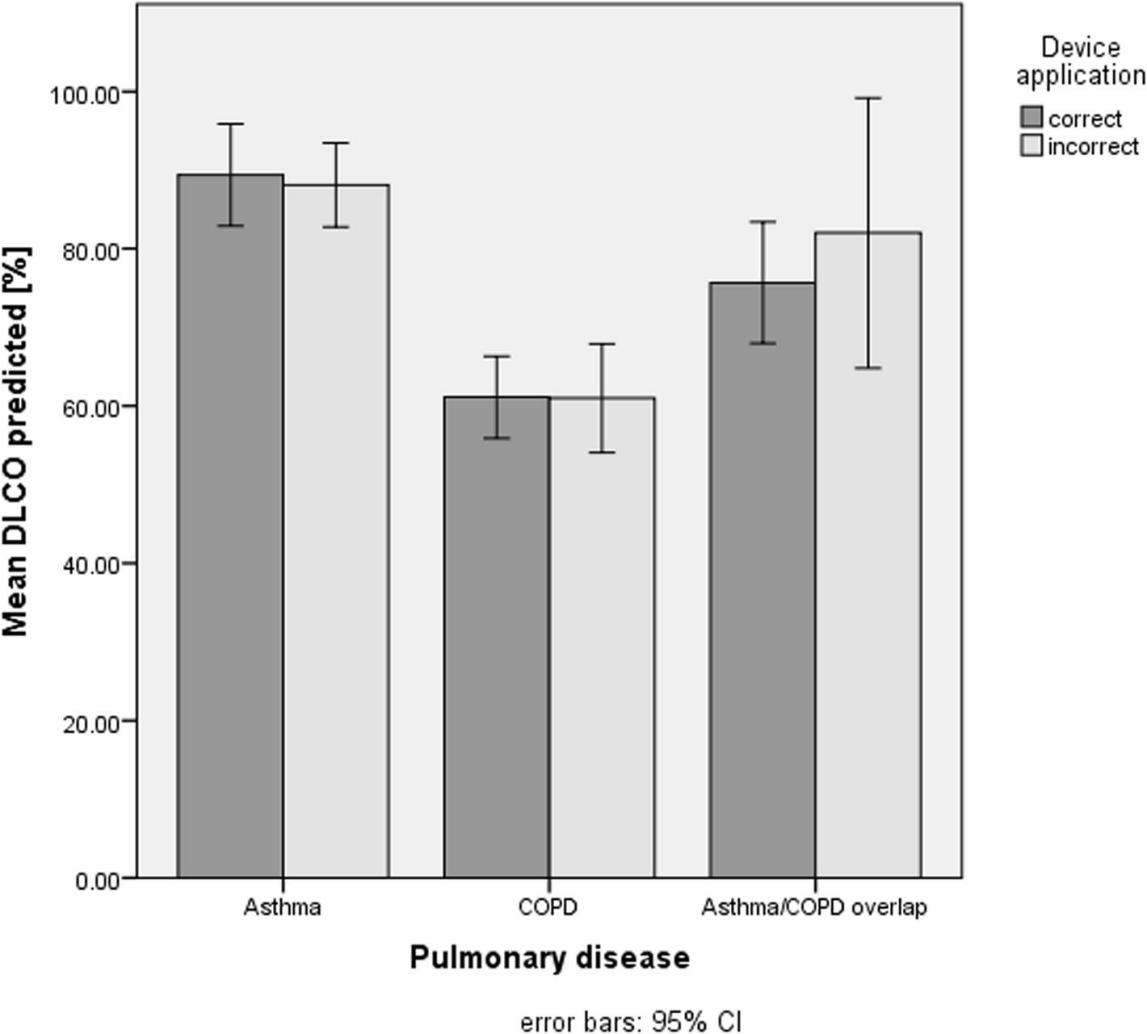
Comparison of the mean DLCO % predicted in patients applying their devices correct and incorrect. DLCO, diffusing capacity of the lung for carbon monoxide

DLCO values between participants with correct and incorrect device application showed no difference neither for patients with asthma nor with for patients with COPD (Fig. 5).

## Discussion

### Main findings

Overall, the application of metered dose inhalers and dry powder inhalers, such as Turbohalers and Breezhaler, showed more incorrect application by the patients while the devices such as Discus and Ellipta® were used more often correctly. In asthma patients, device application had no impact - neither on the ACT score nor on the lung function parameters. However, in COPD patients, incorrect device application had a negative impact on the CAT score. Furthermore, those who applied their devices correctly had a better forced expiratory volume in one second (FEV_1_).

### Patient characteristics

In our study, patient characteristics appeared to be comparable to previous studies with regard to the perception of disease. Thus, 40% of the asthma patients indicated that their disease was not well- or poorly controlled while 30% of the COPD patients stated a high or very high impact of the ailment on their health status. These results are comparable to prior studies by Guénette et al., where 48% of the patients reported uncontrolled asthma [20] and by Dürr et al. where 34% of the patients had uncontrolled asthma at baseline [21]. The mean CAT sum score was 16.7 in COPD patients at baseline, indicating a medium impact on health status – a value also observed in the PHARMACOP study, which examined the effectiveness of a pharmaceutical care programme in patients suffering from COPD [22].

### Device application and different inhaler types

Correct handling of inhaler devices was found to be very type-specific. Metered dose inhalers were more frequently applied in an incorrect way among the study population than dry powder inhalers. The same phenomenon was observed for dry powder inhalers like Turbohaler, Handihaler and Breezhaler. In contrast, the use of the dry powder inhaler Ellipta® was more often correct in the investigated sample.

Frequent application errors identified in the study population were also confirmed by other studies. The results of the CRITIKAL study named the step of “coordination of actuation and inhalation” as one of the main errors in the application process of metered dose inhaler with an error rate of 37% [23].

Similar handling errors with the Handihaler were reported by Kiser et al. [24]. In this study, breath-holding for a sufficient amount of time after inhalation was identified to be performed wrong in 40% of the cases. Even after an intervention, 30% of the Handihaler applications were performed incorrectly.

Since the correct use of an inhaler by patients is directly related to high drug delivery at the target location to the efficacy of the therapy and thereby for sufficient disease control [9, 25, 26], the selection of an adequate inhaler type taking into account the skills and preferences of the individual patient is an important aspect with regard to therapeutic success. This underlines the recommendation by Hodder et al., who stated that the satisfaction and preference of a patient for his inhaler device seems to have a potential impact on the adherence to therapy and consequently on the long-term outcomes of the disease [27].

The good applicability of the Ellipta® device can certainly be explained by the fact, that the application itself is very simple. However, compared to the Discus, there are not many differences regarding the application. Nevertheless, the correct handling of an Ellipta® device seems to be easier. It has to be noted that Ellipta® devices have just been introduced to the market. Therefore, the instructions for correct use provided by a doctor or pharmacist might be more detailed and informative compared to information related to older inhalation devices.

At any rate, the findings from the Adherence-Trial underline the importance of providing a comprehensive introduction to newly prescribed medications and a continuous educational training regarding patient’s recent developments in disease and therapy. This is particularly important in order to ensure that patients are continuously and actively involved in the treatment procedure [21]. Furthermore, these findings reconfirm the recommendation by the Global Initiative for Asthma (GINA) and Global Initiative for Chronic Obstructive Lung Disease (GOLD) guidelines to regularly re-evaluate the correct device application to prevent faulty long-term device use [5, 28].

### Exacerbation history and device application

Participants using their inhaled devices with at least one handling error (minor or critical errors) had significantly more exacerbations in the 12 months before study start compared to participants with a correct handling of their inhaled medication. However, the comparison of participants with at least one critical error and participants with minor errors showed no significant difference with respect to the exacerbation history. These results could also be confirmed by previous studies which demonstrated that device handling errors including critical errors are associated with severe exacerbations [23, 29, 30].

### ACT and CAT scores and correct/incorrect device application at baseline

In asthma patients, the comparison of the ACT sum score as well as the individual ACT questions with the correct/ incorrect device application did not indicate any difference at baseline. This can be explained by the fact that all patients had to be in a stable condition and free of exacerbation for at least one month at the time of their inclusion into the study. Generally, asthma patients show none to very few symptoms during a stable phase of their disease. Moreover, all participants suffering from asthma had an ACT mean sum score of around 20, signifying well-controlled disease condition at that point of time.

However, one could assume that an observation of patients in an acute deterioration phase would indicate a difference when comparing the ACT sum score with the correct/ incorrect device application. Patients applying their inhalation device correctly would be expected to benefit more from the inhaled medication and would therefore show better symptom control compared to patients who use their device incorrectly. Price et al. conducted a real-life study with asthma patients, that showed that there is a difference between the type of inhaler used and the asthma disease outcome. Participants, who applied easier-to-use-inhalers and therefore had higher numbers of correct device applications showed better disease control [31].

A significant difference in the CAT sum score was found when comparing COPD patients with correct and incorrect device application. Patients with an incorrect device application reported a higher impact of their disease on their health status. Subgroup analyses taking into account individual CAT questions revealed significant differences for symptoms like coughing and breathlessness. This may be explained by the fact that patients who apply the inhaler devices incorrectly are not expected to fully benefit from the effect of the prescribed medication, leading to more COPD symptoms like coughing and breathlessness during efforts. Furthermore, uncontrolled symptoms may adversely affect patients´ attitudes towards their medication. If they have the feeling that a therapy is not working, their adherence will be correspondingly low and the patient will no longer inhale the medication [27, 32, 33].

### Lung function parameters and correct/incorrect device application at baseline

Interestingly, no differences were found for FEV_1_ in asthma patients at baseline when comparing correct versus incorrect device application. As mentioned before, the average FEV_1_ was very high at baseline, providing a reasonable explanation for this finding. Nevertheless, this demonstrates once again that asthma patients may have no tangible impairment of their lung function during a stable phase and that even severely impaired lung function may be fully reversible after acute exacerbations.

However, significantly higher FEV_1_ values were found in COPD patients who applied their devices correctly compared to those patients who did not. This finding underlines the results from previous studies revealing that correct and sustained use of inhaled medication is associated with a reduced loss of lung function and an improvement in quality of life [34–37].

### Limitations

This cross-sectional analysis is based on data assessed at the beginning of the Adherence-Trial. We have no information about the course of the lung disease and medication adjustments before inclusion, since these were not recorded. Therefore, results should be interpreted with caution.

Furthermore, since the patients had to be in a stable and exacerbation-free phase four week before inclusion, there is a bias regarding the health status of the study patients. Most patients described good health and good quality of life at baseline. This could lead to overestimation of the patient’s health status and quality of life at the start of the study.

Due to sub analyses using the individual lung diseases (asthma, COPD, asthma-COPD overlap) a limited power must be expected for the investigations which were subdivided in the subclasses, which represents a further limitation.

In addition, the investigator who evaluated the device application of the patients was not blinded to the patient’s QoL scores and lung function parameters, which represents a potential source of bias. In order to diminish this bias, the chronological order of the clinical visits was set in a way that the evaluation of the device application was performed as a first step and after that all the other assessments (lung function tests, questionnaires, etc.) were performed. However, always the same person evaluated the device application, in order to avoid the possibility of interrater variability for the evaluation.

Patients were aware that their inhalation technique was evaluated, which consequently introduces further bias. To minimize these bias, patients were blinded to which steps were defined as critical and that they had to reach a maximal total score to be evaluated as correct.

## Conclusion

To knowledge, this study is one of the first to investigate a population affected by both, asthma and COPD and with no imposed restrictions regarding inhaler devices. Therefore, the application of all available inhaler devices could be investigated and analysed. Due to the wide selection of patients, the present data helps to describe the study population with respect to individual inhalation skills and to get an insight into the current primary care situation looks like. This, enables to derive implications for future care regarding inhalation techniques. This study suggests that regular and comprehensive training of correct inhalation technique is mandatory in patients with chronic lung disease in particular in patients with COPD. Patients who apply their prescribed inhaled medication correctly seem to experience less impact of the disease on their health status and less limitation in their lung function. Moreover, the evaluated data showed that participants who handled their devices correctly had a significantly smaller history of exacerbations compared to participants with device handling errors, which also included critical errors. The findings from this study should encourage health professionals to continuously provide instructions on correct inhalation technique and to re-evaluate the patient’s inhalation technique on a regular basis. By increasing the patient’s responsibility as well as integrating him or her into the treatment process, faulty use of inhalation devices can be prevented in the long term with beneficial effects on signs, symptoms and progression of disease. In order to make a meaningful statement, these results should be confirmed by further analyses that evaluate the disease control, therapy usage and adjustments during the months before study inclusion in more detail. Since the correct application of an inhaler by patients is directly associated with the efficacy of the therapy, the choice of the inhalation devices represents an important step in order to achieve therapeutic success. Therefore, it is proposed that future studies should investigate the preferences and the handling skills of different patient categories in order to ensure a suitable device choice which is individualized according to the patient’s need whenever possible.

## Abbreviations

ACO: Asthma-COPD-Overlap
ACT: Asthma Control Test
BMI: Body mass index
CAT: COPD Assessment Test
COPD: Chronic Obstructive Pulmonary Disease
DLCO: Diffusing capacity of the lung for carbon monoxide
FEV_1_: Forced expiratory volume in one second
FVC: Forced vital capacity
GINA: Global Initiative for Asthma
GOLD: Global Initiative for Chronic Obstructive Lung Disease
ICS: Inhaled corticosteroid
LABA: Long acting beta_2_- agonist
LAMA: Long acting muscarinic antagonist
PY: Pack years
QoL: Quality of life
SABA: Short acting beta_2_ agonist
SAMA: Short acting muscarinic antagonist
SD: Standard deviation
SGRQ: St. George Respiratory Questionnaire
